# Diagnostic Performance of Preoperative Calcitonin and Procalcitonin Tests for Differential Diagnosis of Medullary Thyroid Cancer

**DOI:** 10.3390/diagnostics14161809

**Published:** 2024-08-20

**Authors:** Il Youb Jeong, Hyeok Jun Yun, Seok-Mo Kim, Yongjung Park

**Affiliations:** 1 Department of Laboratory Medicine, Severance Hospital, Yonsei University College of Medicine, Seoul 03722, Republic of Korea; i2runsuga@yuhs.ac; 2 Department of Laboratory Medicine, Gangnam Severance Hospital, Yonsei University College of Medicine, Seoul 06273, Republic of Korea; 3 Thyroid Cancer Center, Institute of Refractory Thyroid Cancer, Department of Surgery, Gangnam Severance Hospital, Yonsei University College of Medicine, Seoul 06273, Republic of Korea; gsyhj@yuhs.ac; 4 Department of Laboratory Medicine, Yongin Severance Hospital, Yonsei University College of Medicine, Yongin 16995, Republic of Korea

**Keywords:** calcitonin, procalcitonin, medullary thyroid cancer, diagnostic performance

## Abstract

Medullary thyroid cancer (MTC) shows a relatively poor prognosis among thyroid cancers. Though calcitonin has been used as a diagnostic marker for MTC, it has disadvantages including poor sample stability and discrepancies among results by assay. This study aimed to compare the usefulness of preoperative calcitonin and procalcitonin (PCT) in the diagnosis of MTC. Serum calcitonin and PCT levels were measured before thyroidectomy from MTC (*n* = 23) and other types of thyroid cancers in patients (*n* = 1308). Diagnostic performances of calcitonin and PCT for discerning MTC were estimated. In a multivariate analysis, preoperative calcitonin level was independently associated with the diagnosis of MTC, whereas PCT was not. Calcitonin and PCT, respectively, exhibited area under the curve values of 0.997 and 0.979 for the diagnosis of MTC, without significant differences. For calcitonin, the sensitivity, specificity, and positive and negative predictive values were 0.957, 0.992, 0.688, and 0.999, respectively, at a cut-off of 7.2 pg/mL. The corresponding values for PCT were 0.913, 0.995, 0.778, and 0.998 at a cut-off of 0.19 ng/mL. Preoperative calcitonin and PCT showed similar diagnostic utility for MTC. Depending on the patient’s clinical status and laboratory environment, these tests can be used as complementary methods for detecting MTC.

## 1. Introduction

Medullary thyroid cancer (MTC) is a malignant tumor of the C cells of the thyroid gland and accounts for approximately 0.6% of Korean thyroid cancer cases and about 1–2% of American cases [1,2]. The 10-year survival rate for MTC is approximately 73%, and it is considered a histologic type of thyroid cancer with a relatively poor prognosis [3]. Factors such as extrathyroidal extension and the stage at diagnosis can influence the prognosis. The primary treatment for MTC is surgery, usually a total thyroidectomy with central lymph node dissection. In some cases, more extensive lymph node dissection may be performed, depending on the stage of the disease. Therefore, early diagnosis and appropriate surgical treatment are essential for a favorable prognosis. Calcitonin and carcinoembryonic antigen (CEA) have been suggested as biochemical markers for MTC in the National Comprehensive Cancer Network and American Thyroid Association (ATA) Guidelines for Management of MTC [2].

Calcitonin, secreted by C cells, has been used as a sensitive and specific marker for the diagnosis and monitoring of MTC. However, it is necessary to process samples quickly to obtain accurate test results, due to the relatively rapid degradation of calcitonin by serum proteases [4]. In addition, calcitonin can exist in a variety of immunoreactive isoforms and fragments [5]. Therefore, the type and composition of isoforms and fragments in an individual can sometimes lead to inaccurate results that are lower than expected and can also vary between assays, which reduces the commutability of results among test principles and methods.

On the other hand, CEA, a non-specific marker for MTC, can be used for the follow-up of MTC patients and is a reliable marker when used to predict disease progression [6,7]. However, elevated CEA levels can also be found in patients with heterophilic antibodies, gastrointestinal inflammatory diseases, benign lung diseases, and non-thyroid malignancies, as well as in smokers [2]. Also, CEA is not sensitive enough to be used for the early detection of the persistence or recurrence of MTC after surgery.

Procalcitonin (PCT) is a precursor of calcitonin, produced by the C cells of the thyroid gland, and its concentration in the bloodstream of healthy adults is very low [8]. The half-life of PCT in the human body is 20–24 h, and it shows excellent in vitro stability when collected as serum or plasma samples [9,10]. There is a strong correlation between PCT and calcitonin concentrations in patients with MTC, and previous studies have reported similar sensitivities in the diagnosis and follow-up of MTC [11,12,13].

The aim of this study was to analyze the levels of calcitonin and PCT determined before thyroidectomy surgery in thyroid cancer patients. Additionally, we compared the diagnostic utility of the tests for the discrimination of MTC from other histologic types of thyroid cancers to suggest how these tests can be used efficiently in clinical practices.

## 2. Materials and Methods

### 2.1. Patients

We conducted a retrospective analysis of the medical records of patients diagnosed with thyroid cancer who underwent thyroidectomy during a 32-month period from January 2021 to August 2023 at Gangnam Severance Hospital, Yonsei University College of Medicine, Seoul, Republic of Korea. The inclusion criteria were adults aged 19 years or older, who underwent surgery for known thyroid nodules and were histologically diagnosed with thyroid cancer before and after surgery, and who had results for both preoperative calcitonin and PCT tests within one week. The final diagnosis of the histologic type of thyroid cancer was determined by specialized pathologists solely based on the pathologic examination, including immunohistochemistry stains on surgical specimens after thyroidectomy, without considering the levels of calcitonin or PCT. Patients were excluded if their PCT levels were high due to concomitant infectious disease or if the histologic type of thyroid cancer was indeterminate on postoperative histologic examination. After excluding 91 cases with uncertain histology, a total of 1331 patients were analyzed and divided into four groups based on the histologic type of thyroid cancer: papillary carcinoma, follicular adenoma/carcinoma, medullary carcinoma, and anaplastic carcinoma.

### 2.2. Laboratory Assays

The serum PCT concentration was measured by a chemiluminescence immunoassay technique, using a VITROS^®^ XT 7600 Integrated System (Ortho Clinical Diagnostics, Raritan, NJ, USA) with a lower limit of detection (LoD) of 0.01 ng/mL and a lower limit of quantitation (LoQ) of 0.03 ng/mL. The assay time of the PCT assay is 24 min and the claimed within-laboratory imprecision is 5.2% CV for a mean PCT concentration of 0.037 ng/mL and between 2.4% CV and 4.0% CV for mean PCT concentrations from 0.093 to 55.2 ng/mL. The serum calcitonin level was measured by a chemiluminescence immunometric assay using an IMMULITE 2000 XPi Immunoassay System (Siemens Healthineers, Erlangen, Germany) with an LoD of 0.1 pg/mL and an LoQ of 2.0 pg/mL. The assay time of the calcitonin assay is 65 min and the claimed within-laboratory imprecision is 15.7% CV for a mean calcitonin level of 11.5 pg/mL and between 3.3% CV and 6.9% CV for mean calcitonin levels from 159.0 to 1628.0 pg/mL. The upper limits of the reference intervals are 8.4 pg/mL for males and 5.0 pg/mL for females. Free thyroxine, thyroid-stimulating hormone, parathyroid hormone, thyroglobulin, and anti-thyroglobulin antibody were assayed by using a cobas e 801 immunoassay analyzer with respective Elecsys reagents (Roche Diagnostics GmbH, Mannheim, Germany). All assays were conducted according to the manufacturer’s instructions.

### 2.3. Statistical Analyses

All statistical analyses in this study were conducted as two-tailed tests with a significance level of 0.05. The statistical analysis was conducted using the Analyse-it Ultimate Edition Ver. 6.15.4 (Analyse-it Software, Ltd., Leeds, UK). Demographic and clinical data were categorized by histologic types and presented by groups. To analyze differences between groups, continuous variables were analyzed using the Mann–Whitney U test or Kruskal–Wallis test, with the Tukey–Kramer paired comparison test used to compensate for alpha error in multiple comparisons. The chi-square test was used to compare the proportions of categorical variables among groups. Binary logistic regression, with the histologic diagnosis of MTC as the dependent variable, and patient age and sex and the results of calcitonin and PCT as the multivariate independent variables, was performed to determine whether the variables were independently associated with the diagnosis of MTC. The diagnostic performance of each test was presented by performing a receiver operating characteristic (ROC) curve analysis to obtain the area under the curve (AUC). For qualitative analysis, the sensitivity, specificity, positive predictive value (PPV), and negative predictive value (NPV) with respective 95% confidence intervals (CIs) were calculated.

## 3. Results

The characteristics of the study subjects are summarized in Table 1. A total of 1331 patients who underwent calcitonin and PCT tests prior to thyroidectomy were analyzed. The patients were classified into four groups based on postoperative tissue pathology: papillary (1244 cases, 93.5%), follicular (48 cases, 3.6%), anaplastic (15 cases, 1.1%), and medullary thyroid cancer (23 cases, 1.7%). The overall proportion of males in total cases was 19.3%, and the proportion of males in MTC and anaplastic thyroid cancer was 39.1% and 53.3%, respectively, which was relatively higher than those of the other two types of thyroid cancers. The levels of calcitonin and PCT were elevated in MTC but did not increase in other thyroid cancers.

The multivariate analysis in Table 2 shows that a 1 pg/mL increase in calcitonin was independently associated with MTC with an odds ratio (OR) of 1.305; sex, age, and PCT level did not show significant associations.

**Figure 1:**
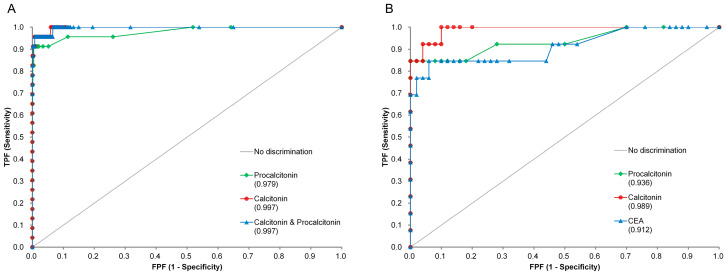
Diagnostic performances of calcitonin, procalcitonin (PCT), and carcinoembryonic antigen (CEA) for the discrimination of medullary thyroid cancer (MTC) from other histologic types of thyroid cancer. Calcitonin, PCT, and their combination showed excellent diagnostic performance for MTC diagnosis (23 cases and 1308 controls) with the area under the curve (AUC) values greater than 0.97, and the AUC values of the tests were similar to one another (**A**). In the subgroup of patients with CEA results (13 cases and 50 controls), the AUC values of calcitonin, PCT, and CEA were 0.989, 0.936, and 0.912, respectively, but they were not statistically different (**B**).

The results of the ROC curve analysis for the diagnosis of MTC are shown in Table 3 and Figure 1. When comparing 23 cases of MTC and 1308 cases of other thyroid cancers, the AUC of calcitonin alone, PCT alone, and the calcitonin and PCT combinations were 0.997, 0.979, and 0.997, respectively. However, there was no significant difference when comparing the AUC of calcitonin with those of other parameters. Of the total 1331 patients, 63 underwent preoperative CEA testing, including 13 with MTC and 50 with other thyroid cancers. When the ROC curve analysis was performed on the patients with CEA results, the AUC values of calcitonin, PCT, and CEA were 0.989, 0.936, and 0.912, respectively.

Table 4 compares the sensitivity, specificity, PPV, and NPV of calcitonin and PCT in the diagnosis of MTC. When the cut-off value was set at the maximum, Youden’s index, the sensitivity, specificity, and PPV of calcitonin were 0.957, 0.992, and 0.688, respectively; and those of PCT were 0.913, 0.995, and 0.778.

In the 22 MTC patients for whom the tumor size could be analyzed in the pathology results, we calculated the correlation coefficient between the maximum diameter of the MTC and each test (Table 5). The size of thyroid cancer nodules ranged from 0.3 cm to 4.5 cm, with Spearman’s rank correlation coefficients of 0.628, 0.599, and 0.575 for calcitonin, PCT, and CEA, respectively, indicating moderate positive correlations.

## 4. Discussion

In this retrospective study, a total of 1331 patients with thyroid cancer were included to assess the usefulness of preoperative calcitonin and PCT for differential diagnosis of MTC. Patients were divided into four groups: papillary, follicular, medullary, and anaplastic thyroid cancer, which were similar to the previously known proportions of thyroid cancer [1,14]. In addition, our results showed a higher proportion of male patients with medullary and anaplastic thyroid cancer compared to other thyroid cancers, which is consistent with previous studies [1]. The relatively higher proportion of males with MTC compared to other thyroid cancers may be related to the two times higher C cell density in males than in females [15].

In our results of the multivariate analysis, only calcitonin was independently associated with MTC (Table 2). This would result from the insufficient statistical power due to the small number of MTC cases, or it could be because PCT and calcitonin act as a statistical confounder with each other. In our data, Spearman’s correlation coefficient between calcitonin and PCT was 0.292 (95% CI 0.241 to 0.342, *p* < 0.0001). On the other hand, the ROC curve analysis indicated that calcitonin, PCT, and CEA all showed significant values in the differential diagnosis of MTC. When comparing PCT alone to calcitonin alone, the AUC values of the two tests were not statistically different. However, elevated calcitonin levels can be observed in conditions other than MTC, such as acute trauma, chronic pulmonary diseases, chronic renal failure, hypercalcemia, hypergastrinemia, inhalation injury, other neuroendocrine tumors, pseudohypoparathyroidism, and thyroiditis [16,17,18,19]. In the present study, among the 1308 non-MTC patients, there were 19 with calcitonin levels above the upper limit of the reference interval, while PCT levels were not greater than 0.14 ng/mL in 18 of these patients. When applying the respective cut-off values showing the best diagnostic accuracy for each assay (7.2 pg/mL for calcitonin and 0.19 ng/mL for PCT), one case among 23 MTC cases was positive only for calcitonin, another case was negative for both calcitonin and PCT, and the rest of the 21 patients were positive for both calcitonin and PCT. This result suggests that the sensitivity of calcitonin for MTC diagnosis would be better than that of PCT, while PCT would have better specificity than calcitonin. When considering the purpose of the assays in different clinical situations, an integrated approach using multiple tests would improve diagnostic accuracy. Meanwhile, calcitonin can be degraded by serum proteases; thus, samples should be frozen to ensure accurate analytical results when immediate testing is not possible. On the other hand, samples for PCT assay can be stored for 24 h at room temperature or 48 h at refrigerated temperatures. Therefore, PCT assays would have advantages in terms of not only a shorter assay time and better precision performance but also the ease of handling the samples in general clinical circumstances.

Determining the optimal cut-off value is important for clinical applications. The interpretation of calcitonin levels is difficult because the reference interval for calcitonin is gender-specific, and method-specific, and may be elevated in benign thyroid diseases and certain extrathyroidal conditions. According to the existing literature, a cut-off value of 10 pg/mL yields a sensitivity between 0.92 and 1.00, and a specificity between 0.94 and 0.99 [20]. In addition, the ATA guidelines recommend imaging studies when the basal serum calcitonin level is 150 pg/mL or higher [2]. Studies using different cut-offs for males and females have shown sensitivities of 0.53 to 1.00 and specificities of 0.95 to 1.00 at cut-offs of 34 to 68 pg/mL in males, and sensitivities of 0.76 to 1.00 and specificities of 0.88 to 1.00 at cut-offs of 18.7 to 35 pg/mL in females [21]. For PCT, previous studies suggested cut-off values of 0.07 to 0.155 ng/mL, with sensitivities of 0.86 to 1.00 and specificities of 0.90 to 1.00 [22,23,24,25,26,27]. When comparing calcitonin results by different assay methods, there is a low level of agreement [28,29]. In contrast, PCT has a higher degree of agreement among assays [30,31]. Therefore, establishing a general cut-off value may be more useful for PCT than for calcitonin in a clinical setting.

Calcitonin levels showed a maximum Youden’s index at a cut-off of 7.2 pg/mL in our data. This value is lower than the upper reference limit for males of the assay used in this study, suggesting that MTC cannot be excluded even if the calcitonin level is within the normal reference limit. In this study, calcitonin levels were within the reference interval in 2 out of 23 MTC patients. The PPV of calcitonin was 0.688, which is lower than the PPV reported in previous studies (0.85–1.0) [21]. This difference may be due to the lower cut-off value used in this study compared to other studies.

Among the twenty-three MTC patients, one had both calcitonin and PCT levels below the cut-off, presenting a small tumor size of 0.3 cm. In a previous study, a strong positive correlation (R^2^ = 0.75) between calcitonin and PCT levels was reported [26]. Given the moderate correlation between tumor size and calcitonin or PCT levels in our results, the possibility of MTC cannot be ruled out on the basis of either level alone, especially in cases with a small thyroid nodule.

This study has several limitations, primarily the small number of patients analyzed for MTC. We included all eligible cases of more than 1300 thyroid cancer patients; however, due to the low incidence of MTC in Korea, only a limited number of MTC cases could be analyzed. This limitation would also lead to statistically overfitted results, but unfortunately, cross-validation could not be performed in this study. Extending the study duration and increasing the number of patients analyzed will enhance the statistical robustness of the results. The second point is that this study focused on patients who were histologically diagnosed with thyroid cancer. Therefore, caution is required when interpreting calcitonin and PCT tests conducted for diagnostic purposes before confirming thyroid cancer. For instance, when testing for thyroid nodular disease, using the same cut-off values may yield a lower PPV for diagnosing MTC. In addition, since this study showed a correlation between MTC nodule size and the results of each test, further studies are needed to determine whether these test results are associated with the prognosis of MTC patients.

In conclusion, calcitonin and PCT showed similar diagnostic performance in the differential diagnosis of MTC. Depending on the patient’s clinical condition and laboratory resources, these tests can be used as complementary methods for detecting MTC in patients suspected of thyroid cancer due to thyroid nodules.

## Figures and Tables

**Table 1:** Characteristics of study subjects.

Parameter	Papillary * (*n* = 1244)	Follicular ** (*n* = 48)	Anaplastic (*n* = 15)	Medullary (*n* = 23)	*p*-Value
Age (years)	40 (34 to 48)	41 (33 to 48)	69 (61 to 70)	45 (39 to 61)	<0.0001
Sex (N of male, %)	234, 18.8%	6, 12.5%	8, 53.3%	9, 39.1%	0.0003
Preoperative laboratory results					
Free thyroxine (ng/dL)	1.3 (1.1 to 1.4)	1.3 (1.2 to 1.4)	1.3 (1.1 to 1.4)	1.3 (1.2 to 1.3)	0.8967
Thyroid-stimulating hormone (μIU/mL)	1.86 (1.24 to 2.78)	1.97 (0.84 to 2.45)	1.65 (0.95 to 4.34)	1.74 (1.20 to 3.39)	0.7878
Thyroglobulin (ng/mL)	13.65 (6.55 to 26.50)	66.15 (25.13 to 292.92)	37.70 (6.86 to 106.10)	10.40 (3.12 to 20.87)	<0.0001
Anti-thyroglobulin antibody (IU/mL)	14.5 (12.9 to 39.7)	14.7 (13.1 to 28.5)	20.0 (12.8 to 327.2)	14.4 (13.5 to 16.0)	0.6468
Parathyroid hormone (pg/mL)	37.6 (29.7 to 47.6)	40.3 (29.1 to 50.9)	22.4 (13.4 to 47.3)	32.3 (25.0 to 50.9)	0.0589
Serum calcium (mg/dL)	9.2 (9.0 to 9.4)	9.3 (9.0 to 9.5)	9.5 (9.1 to 9.7)	9.0 (8.9 to 9.5)	0.0625
Calcitonin (pg/mL)	<2.0 (<2.0 to <2.0)	<2.0 (<2.0 to <2.0)	<2.0 (<2.0 to <2.0)	310.0 (51.8 to 683.3)	<0.0001
Procalcitonin (ng/mL)	0.04 (<0.03 to 0.05)	0.04 (<0.03 to 0.04)	0.05 (0.04 to 0.06)	1.26 (0.53 to 3.53)	<0.0001
Genetic abnormality (positive/tested, %)					
*BRAF* V600 mutation	992/1219, 81.4%	2/12, 16.7%	5/14, 35.7%	3/6, 50.0%	
*TERT* promoter mutation	13/1222, 1.1%	1/11, 9.1%	11/14, 78.6%	0/6, 0.0%	
*RET* gene mutation	0/9, 0.0%	−	0/14, 0.0%	5/13, 38.5%	
Surgical specimen					
Maximum diameter of tumor (cm)	0.8 (0.5 to 1.2)	3.0 (1.8 to 4.3)	4.0 (3.2 to 6.0)	1.5 (0.9 to 2.2)	<0.0001
Lymph node metastasis (positive, %)	503, 40.4%	1, 2.1%	8, 53.3%	13, 56.5%	<0.0001
Immunohistochemistry stain (positive/tested, %)					
MARS1	1015/1017, 99.8%	15/15, 100.0%	11/11, 100.0%	6/6, 100.0%	
HBME-1	40/42, 95.2%	4/22, 18.2%	8/10, 80.0%	0/1, 0.0%	
CD56 (SCLC)	22/36, 61.1%	19/22, 86.4%	1/2, 50.0%	4/4, 100.0%	
CK19	29/35, 82.9%	6/22, 27.3%	2/2, 100.0%	0/1, 0.0%	
Calcitonin	0/1, 0.0%	−	0/1, 0.0%	22/22, 100.0%	
Congo red	−	−	−	9/12, 75.0%	

Abbreviations: MARS1, methionyl-tRNA synthetase 1; HBME-1, Hector Battifora mesothelial-1; SCLC, small cell lung carcinoma; CK19, cytokeratin 19. Values are shown as median (1st to 3rd quartiles). One case of poorly differentiated thyroid carcinoma was excluded from this table. * Conventional or follicular variant papillary carcinoma. ** Includes follicular adenoma (*n* = 31), follicular carcinoma (*n* = 12), diffuse follicular hyperplasia (*n* = 2), and non-invasive follicular thyroid neoplasm with papillary-like nuclear features (NIFTP) (*n* = 3).

**Table 2:** Results of multivariate analysis using binary logistic regression for discriminating MTC from other histologic types of thyroid cancers.

Variable	Odds Ratio (95% CI)	*p*-Value
Male	0.314 (0.034 to 2.907)	0.3075
Age (per 1 year increase)	1.090 (0.997 to 1.193)	0.0588
Calcitonin (per 1 pg/mL increase)	1.305 (1.138 to 1.496)	<0.0001
Procalcitonin (per 0.1 ng/mL increase)	1.698 (0.897 to 3.214)	0.1040

Abbreviations: MTC, medullary thyroid cancer; CI, confidence interval.

**Table 3:** Results of ROC curve analysis for discriminating MTC from other histologic types of thyroid cancers.

Parameter	Dataset	AUC (95% CI)	*p*-Value	*p*-Value for Difference *
Calcitonin	23 cases vs. 1308 controls	0.997 (0.992 to 1.002)	<0.0001	−
Procalcitonin	23 cases vs. 1308 controls	0.979 (0.945 to 1.012)	<0.0001	0.2144
Calcitonin + procalcitonin	23 cases vs. 1308 controls	0.997 (0.991 to 1.002)	<0.0001	0.3469
Calcitonin	13 cases vs. 50 controls	0.989 (0.971 to 1.008)	<0.0001	−
Procalcitonin	13 cases vs. 50 controls	0.936 (0.841 to 1.032)	<0.0001	0.1955
CEA	13 cases vs. 50 controls	0.912 (0.800 to 1.023)	<0.0001	0.1294

Abbreviations: ROC, receiver operating characteristics; MTC, medullary thyroid cancer; AUC, area under the curve; CI, confidence interval; CEA, carcinoembryonic antigen. * *p*-values were calculated by comparing the AUC of calcitonin with those of other parameters.

**Table 4:** Diagnostic performance of each test for discriminating MTC from other histologic types of thyroid cancers.

Assay	Condition	Cut-Off	Youden’s Index	Sensitivity (95% CI)	Specificity (95% CI)	PPV (95% CI) *	NPV (95% CI) *
Calcitonin (pg/mL)	Maximum Youden’s index	>7.2	0.949	0.957 (0.790 to 0.992)	0.992 (0.986 to 0.996)	0.688 (0.541 to 0.804)	0.999 (0.995 to 1.000)
	At ~95% specificity	>3.4	0.906	0.957 (0.790 to 0.992)	0.950 (0.936 to 0.960)	0.250 (0.206 to 0.300)	0.999 (0.995 to 1.000)
Procalcitonin (ng/mL)	Maximum Youden’s index	>0.19	0.908	0.913 (0.732 to 0.976)	0.995 (0.990 to 0.998)	0.778 (0.609 to 0.887)	0.998 (0.994 to 1.000)
	At ~95% specificity	>0.06	0.861	0.913 (0.732 to 0.976)	0.948 (0.935 to 0.959)	0.236 (0.192 to 0.287)	0.998 (0.994 to 1.000)

Abbreviations: MTC, medullary thyroid cancer; CI, confidence interval; PPV, positive predictive value; NPV, negative predictive value. * When the prevalence of MTC is 1.7%.

**Table 5:** Correlation between maximum diameter (cm) of MTC and each test.

Test	N	Spearman’s *r_s_* (95% CI)	*p*-Value
Calcitonin (pg/mL)	22	0.628 (0.269 to 0.834)	0.0017
Procalcitonin (ng/mL)	22	0.599 (0.225 to 0.819)	0.0032
CEA (ng/mL)	12	0.575 (−0.017 to 0.869)	0.0503

Abbreviations: MTC, medullary thyroid cancer; CI, confidence interval; CEA, carcinoembryonic antigen.

## Data Availability

The data presented in this study are available upon request from the corresponding author due to the fact that the data are the property of the institution and cannot be shared without permission from the institution.
